# Supplementary material for: Clinical and genomic features of Lynch syndrome differ by tumor site and disease spectrum
Source: Nat Commun. 2025 Nov 19;16:10179. doi: 10.1038/s41467-025-65164-6 (PMC12630928; doi:10.1038/s41467-025-65164-6)
Supplement: Supplementary file 3 — Reporting Summary [file 41467_2025_65164_MOESM3_ESM.pdf]

nature portfolio

Corresponding author(s): Zhaobang Tan and Xing Zhang

Last updated by author(s): Sep 9, 2025

Reporting Summary

Nature Portfolio wishes to improve the reproducibility of the work that we publish. This form provides structure for consistency and transparency in reporting. For further information on Nature Portfolio policies, see our Editorial Policies and the Editorial Policy Checklist.

Statistics

For all statistical analyses, confirm that the following items are present in the figure legend, table legend, main text, or Methods section.

n/a	Confirmed	
☐	☒	The exact sample size (*n*) for each experimental group/condition, given as a discrete number and unit of measurement
☐	☒	A statement on whether measurements were taken from distinct samples or whether the same sample was measured repeatedly
☐	☒	The statistical test(s) used AND whether they are one- or two-sided<br>*Only common tests should be described solely by name; describe more complex techniques in the Methods section.*
☐	☒	A description of all covariates tested
☐	☒	A description of any assumptions or corrections, such as tests of normality and adjustment for multiple comparisons
☐	☒	A full description of the statistical parameters including central tendency (e.g. means) or other basic estimates (e.g. regression coefficient) AND variation (e.g. standard deviation) or associated estimates of uncertainty (e.g. confidence intervals)
☐	☒	For null hypothesis testing, the test statistic (e.g. *F*, *t*, *r*) with confidence intervals, effect sizes, degrees of freedom and *P* value noted<br>*Give P values as exact values whenever suitable.*
☒	☐	For Bayesian analysis, information on the choice of priors and Markov chain Monte Carlo settings
☒	☐	For hierarchical and complex designs, identification of the appropriate level for tests and full reporting of outcomes
☒	☐	Estimates of effect sizes (e.g. Cohen's *d*, Pearson's *r*), indicating how they were calculated

Our web collection on statistics for biologists contains articles on many of the points above.

Software and code

Policy information about availability of computer code

Data collection	No software was used for data collection.
Data analysis	R version 4.3.1 and Excel were used for all analyses. The top mutated somatic genes and germline variant genes were analyzed and visualized utilizing the R package maftools and ComplexHeatmap. Other figures were generated using the R packages ggplot2 and Excel. The differences between the continuous variables were analyzed using the Wilcoxon test, while the chi-square test was chosen for the large sample analysis. The differences between different groups of the matrix pie charts were generated through Fisher's exact test. A P value < 0.05 was considered to indicate statistical significance.

For manuscripts utilizing custom algorithms or software that are central to the research but not yet described in published literature, software must be made available to editors and reviewers. We strongly encourage code deposition in a community repository (e.g. GitHub). See the Nature Portfolio guidelines for submitting code & software for further information.

Data

Policy information about availability of data

All manuscripts must include a data availability statement. This statement should provide the following information, where applicable:

- Accession codes, unique identifiers, or web links for publicly available datasets
- A description of any restrictions on data availability
- For clinical datasets or third party data, please ensure that the statement adheres to our policy

The genomic raw sequence data generated in this study have been deposited in the Genome Sequence Archive (Genomics, Proteomics & Bioinformatics 2025) in

National Genomics Data Center (Nucleic Acids Res 2025), China National Center for Bioinformation / Beijing Institute of Genomics, Chinese Academy of Sciences (GSA-Human: HRA013167) that are publicly accessible at https://ngdc.cncb.ac.cn/gsa-human. The raw sequence data are available under restricted access for the files containing potentially identifiable human genetic information, Chinese regulations on human genetic resources require that access be controlled; consequently, only bona-fide investigators from academic or other non-profit institutions may obtain the dataset for non-commercial research that is consistent with the participants' consent, and any attempt to re-identify individuals is prohibited. Researchers who wish to access the data must log in to the GSA-Human portal, submit an online controlled-access application that includes a brief research plan, local ethical-approval documentation, and a signed institutional Data Transfer Agreement, and are encouraged to address any preliminary questions to the corresponding author, Xing Zhang. The GSA-Human Data Access Committee will evaluate complete requests and issue a decision within ten working days; for approved projects, the download credentials will remain active for 1year. The germline mutation data of TCGA database used in this study are available in Cancer Genome Atlas (https://www.cell.com/cell/fulltext/S0092-8674(18)30363-5). The remaining data are available in the Article, Supplementary Information, or Source Data file. Source data are provided with this paper.

Research involving human participants, their data, or biological material

Policy information about studies with human participants or human data. See also policy information about sex, gender (identity/presentation), and sexual orientation and race, ethnicity and racism.

Reporting on sex and gender	Neither sex nor gender was considered in the study design, since the primary focus of this study was unrelated to sex or gender. All adult participants or guardians provided written informed consent. Participants received no compensation. Among the 228 enrolled patients, 132 were male and 96 were female, with a median age of 52.5 years (range: 0–79).
Reporting on race, ethnicity, or other socially relevant groupings	Analyses performed in this study are based on disease and cancer types. Race, ethnicity, or other socially revevant groupings are not involved.
Population characteristics	A total of 238 samples from 228 patients were enrolled and divided into 3 groups according to the tumor location and tumor spectrum of the LS: the CNS LS-related tumor group (n=68), the Non-CNS LS-related tumor group (n=117), and the Non-CNS LS-unrelated tumor group (n=53). The clinical characteristics of the three groups are shown in Table 1. There were significant differences in age among the three groups, with the age of the CNS LS-related tumor group being lower than that of the other two groups (p< 0.001). Germline variant gene (p< 0.001) and MSI status (p< 0.001) were also significantly different, but there was no significant difference in gender distribution among the three groups.
Recruitment	Pan-cancer samples from the Simceredx database harboring heterozygous pathogenic or likely pathogenic (P/LP) variants in at least one of the MMR genes (MLH1, MSH2, MSH6, and PMS2).
Ethics oversight	This study was approved by the Ethical committee of Xijing Hospital of Digestive Diseases, Fourth Military Medical University (XJLL-KY-20252354) and the Institutional Review Board of Nanjing Simcere Medical Laboratory Science (NSML-IRB-202406-MS03).

Note that full information on the approval of the study protocol must also be provided in the manuscript.

Field-specific reporting

Please select the one below that is the best fit for your research. If you are not sure, read the appropriate sections before making your selection.

- [x] Life sciences
- [ ] Behavioural & social sciences
- [ ] Ecological, evolutionary & environmental sciences

For a reference copy of the document with all sections, see nature.com/documents/nr-reporting-summary-flat.pdf

Life sciences study design

All studies must disclose on these points even when the disclosure is negative.

Sample size	Sample size was not determined before the study. Cohort in this retrospective study included a total of 238 samples from 228 patients.
Data exclusions	A sample from one patient with cancer of unknown primary origin was excluded.
Replication	A total of 238 samples from 228 patients were enrolled and divided into 3 groups according to the tumor location and tumor spectrum of the LS: the CNS LS-related tumor group (n=68), the Non-CNS LS-related tumor group (n=117), and the Non-CNS LS-unrelated tumor group (n=53), with each sample representing a single biological replicate.
Randomization	Not relevant to our study. We did not randomize any patients or samples to any groups. All group assignments of samples were only based on the tumor location and tumor spectrum.
Blinding	Not applicable to this study. We designed a retrospective study. All group assignments of samples were only based on the tumor location and tumor spectrum.

Reporting for specific materials, systems and methods

We require information from authors about some types of materials, experimental systems and methods used in many studies. Here, indicate whether each material, system or method listed is relevant to your study. If you are not sure if a list item applies to your research, read the appropriate section before selecting a response.

Materials & experimental systems

n/a	Involved in the study
☒	☐ Antibodies
☒	☐ Eukaryotic cell lines
☒	☐ Palaeontology and archaeology
☒	☐ Animals and other organisms
☐	☒ Clinical data
☒	☐ Dual use research of concern
☒	☐ Plants

Methods

n/a	Involved in the study
☒	☐ ChIP-seq
☒	☐ Flow cytometry
☒	☐ MRI-based neuroimaging

Clinical data

Policy information about clinical studies

All manuscripts should comply with the ICMJE guidelines for publication of clinical research and a completed CONSORT checklist must be included with all submissions.

Clinical trial registration	Not applicable for this retrospectively analyzed study.
Study protocol	Not applicable, not a clinical trial.
Data collection	The data of 238 final enrolled samples from 228 patients were collected.
Outcomes	Not applicable for this retrospectively analyzed study.

Plants

Seed stocks	*Report on the source of all seed stocks or other plant material used. If applicable, state the seed stock centre and catalogue number. If plant specimens were collected from the field, describe the collection location, date and sampling procedures.*
Novel plant genotypes	*Describe the methods by which all novel plant genotypes were produced. This includes those generated by transgenic approaches, gene editing, chemical/radiation-based mutagenesis and hybridization. For transgenic lines, describe the transformation method, the number of independent lines analyzed and the generation upon which experiments were performed. For gene-edited lines, describe the editor used, the endogenous sequence targeted for editing, the targeting guide RNA sequence (if applicable) and how the editor was applied.*
Authentication	*Describe any authentication procedures for each seed stock used or novel genotype generated. Describe any experiments used to assess the effect of a mutation and, where applicable, how potential secondary effects (e.g. second site T-DNA insertions, mosiacism, off-target gene editing) were examined.*
