# Supplementary material for: Clinical and genomic features of Lynch syndrome differ by tumor site and disease spectrum
Source: Nat Commun. 2025 Nov 19;16:10179. doi: 10.1038/s41467-025-65164-6 (PMC12630928; doi:10.1038/s41467-025-65164-6)
Supplement: Supplementary file 4 — Description of Additional Supplementary Files [file 41467_2025_65164_MOESM4_ESM.pdf]

Description of Additional Supplementary Files

File name: Supplementary Data 1

Description: The incidence ratio of each cancer type in our cohort and TCGA cohort.

File name: Supplementary Data 2

Description: The counts and statistical results of the MSI status corresponding to different genes in each group.

File name: Supplementary Data 3

Description: The gene lists for each panel.
