# Supplementary material for: Clinical and genomic features of Lynch syndrome differ by tumor site and disease spectrum
Source: Nat Commun. 2025 Nov 19;16:10179. doi: 10.1038/s41467-025-65164-6 (PMC12630928; doi:10.1038/s41467-025-65164-6)
Supplement: Supplementary file 1 — Supplementary Information [file 41467_2025_65164_MOESM1_ESM.pdf]

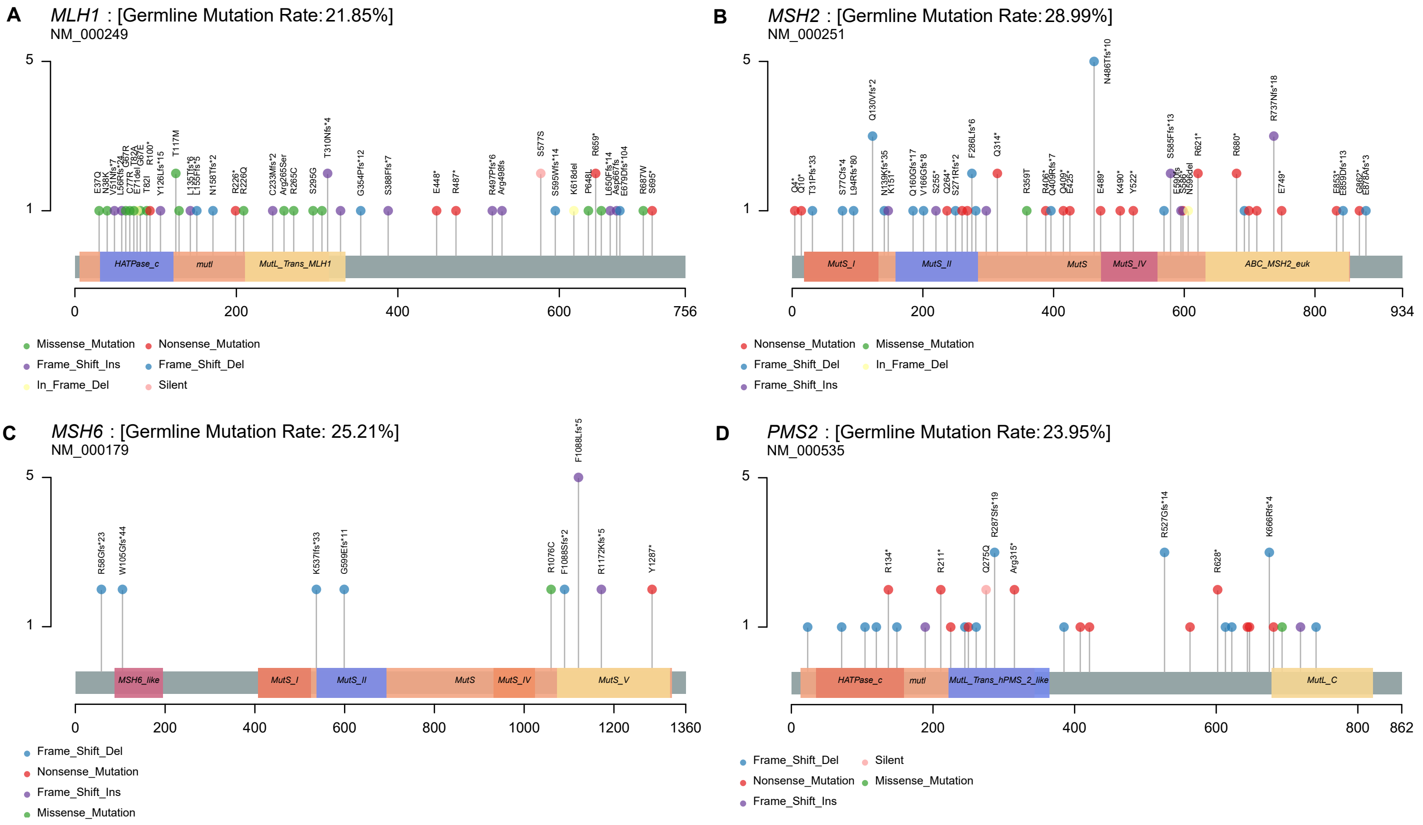

Supplementary Figure 1 Lollipop plot showingthe distribution of MMR gene mutations in protein domains among the total sample.

(A-D) Lollipop plot showing no hotspot mutations and loss-of-function (LOF) mutations in the *MLH1* (A), *MSH2* (B), *MSH6* (C), and *PMS2* (D) genes. The color of the lollipop signifies the type of mutation, while the gray bar indicates the precise location of amino acids, and its height represents the number of variants at those positions. Source data are provided as a Source Data file.
